# Effects of online mindfulness-based interventions on mental and physical health outcomes in cancer patients: A systematic review and meta-analysis of randomized controlled trials

**DOI:** 10.1097/MD.0000000000041870

**Published:** 2025-03-21

**Authors:** Lichun Xu, Aixuan Guan, Yuxin Huang

**Affiliations:** a Department of Nursing, Zhongshan Hospital Affiliated to Xiamen University, Xiamen, China; b Department of Respiratory and Critical Care Medicine, Longyan First Hospital Affiliated to Fujian Medical University, Longyan, China.

**Keywords:** cancer, meta-analysis, mindfulness, online, randomized controlled trial, systematic review

## Abstract

**Background:**

To determine whether online mindfulness-based interventions (MBIs) help improve the physical and mental health of cancer patients, we conducted a systematic review and meta-analysis of relevant randomized controlled trials (RCTs).

**Methods:**

We searched PubMed, Web of Science, Scopus, Embase, Cochrane, Medline, and CINAHL databases for RCTs published up to April 31, 2023. Two reviewers selected and critically appraised the trials and extracted the data. Fourteen RCTs met the inclusion criteria.

**Results:**

A total of 14 RCTs with 1316 participants were part of this meta-analysis. The results showed that the online MBI was effective in improving the anxiety level (standardized mean difference [SMD] = −0.30, 95% confidence interval [CI] (−0.59, −0.01), *P* = .04), stress [SMD = −0.65, 95% CI (−1.23, −0.07), *P* = .03], quality of life [SMD = 0.33, 95% CI (0.17, 0.50), *P* < .0001], sleep quality [SMD = −0.38, 95% CI (−0.65, −0.10), *P* = .007], and fatigue severity [weighted mean difference (WMD) = −3.81, 95% CI (−6.11, −1.51), *P* = .001] in cancer patients. Not shown to affect depression [SMD = −0.19, 95% CI (−0.54, 0.17), *P* = .30].

**Conclusion:**

Online MBIs may be effective in the reduction of psychological distress and other symptoms in cancer patients. However, in view of the limitations of the current study, more rigorously designed RCTs will be needed in the future.

## 1. Introduction

Cancer is the leading cause of illness and death in China and the developed world.^[1,2]^ As the world’s population ages, the incidence of cancer is increasing worldwide, and the number of cancer patients is expected to reach 28.4 million worldwide in 2040, a 47% increase from 2020, posing a serious threat to human health.^[3,4]^ In addition, cancer mortality rates are declining in most parts of the world due to the rapid development of technological tools for cancer treatment.^[5]^ Based on these superior cancer treatment techniques, survival rates of cancer patients have been increased.

Noting that cancer diagnosis and treatment are extremely stressful experiences that can lead patients to endure severe physical or psychological distress, the increasing survival rates offer new rehabilitation problems.^[6]^ For example, 54% to 78% of breast cancer patients report sleep disturbances during treatment,^[7]^ and 70% to 80% report psychological distress.^[8]^ Their quality of life is significantly impacted by such symptom burdens.^[6]^ In particular, the COVID-19 pandemic over the past 3 years has led to serious disturbances in the mental and physical health of cancer patients due to isolation from therapists during quarantine. There is evidence that pandemics have a dramatic impact on the mental health of cancer patients, with a significant increase in psychiatric symptoms among patients during isolation.^[9]^ As a matter of fact, the quality of life of cancer patients is deteriorating during the COVID-19 state of emergency.^[10]^ Therefore, taking appropriate psychosocial interventions decreasing these mental disorders and promoting physical health among cancer patients is significant.

Over the past few years, research indicated that mindfulness-based interventions (MBIs) have gained popularity among cancer patients, and many different applications of MBIs to help deal with patients’ mental health during as well as after cancer treatment.^[11,12]^ Mindfulness is defined as a method of mental training in which the focus of attention is on the present moment.^[13]^ In 1982, the first clinical application of mindfulness, derived from Buddhist thought, began to treat chronic pain.^[14]^ Since then, MBIs have continued to evolve. They have been incorporated into a variety of mental health therapies, including Mindfulness-Based Stress Reduction (MBSR), Mindfulness-Based Cognitive Therapy (MBCT), Dialectical Behavior Therapy, and Acceptance and Commitment Therapy.^[15]^ Of these, the 2 MBI programs that have been most widely adopted are MBSR and MBCT. MBIs, which focus attention through meditation exercises, yoga, group discussions, and didactic teaching, have been shown to be effective in reducing some common mental and physical health problems in the recovery process of cancer patients.^[6,16,17]^

Because of the rapid advancement of information technology, a number of online platforms, including online MBIs, have been used to deliver mental health interventions. Mental health interventions have been delivered using online platforms, including online MBIs.^[18]^ Particularly during the COVID-19 pandemic, segregation, closure, and social alienation limited face-to-face intervention.^[19]^ Compared to traditional face-to-face approaches, online mental health interventions have several advantages: (1) online interventions can be conveniently continued at home; (2) participants can practice at any time and it is easy to join; (3) online interventions can protect the privacy of participants; and (4) they are more cost-effective. In addition, 1 study found that online formats for mindfulness meditation interventions were more popular with cancer patients than face-to-face interventions.^[20]^

In some prior RCT meta-analyses,^[21,22]^ the effectiveness of online MBIs in lowering mental health issues has been assessed, and some other previous meta-analyses focusing on university students have suggested that online MBIs may be effective in improving the mental health of university students.^[23]^ A review and meta-analysis by Liu, Z et al^[21]^ examined 9 RCTs to investigate the effectiveness of online MBIs for improving mental health in patients with physical health problems. Online MBIs led to improvements of some effect size in depression, anxiety, stress and mindfulness. The effectiveness of online MBI for cancer patients, however, is unclear. To bridge the relevant elements in the literature, we extended the review by Liu Z et al.^[21]^ To focus on mental health outcomes in the cancer population. Because studies of populations with the same disease may lead to more consistent conclusions particular to those groups, a tighter focus on disease populations was chosen. Since, physical health conditions may affect mental health, our secondary aim was to explore the effects of online MBIs on physical health.

Therefore, we write this article with the following aims: (1) to review the evidence for the effectiveness of online MBIs for psychological distress in cancer patients. (2) To statistically summarize the effectiveness of reported online MBIs on common mental health problems such as anxiety, depression and stress. (3) Examine effects on a range of secondary outcomes, namely cancer-related quality of life and a range of individual physical symptoms commonly experienced by cancer patients and survivors, including sleep disturbance and fatigue severity. (4) Attempted to determine the quality of this evidence.

## 2. Materials and methods

### 2.1. Study registration

This protocol for a systematic review and meta-analysis was preregistered on the International Prospective Register of Systematic Reviews (PROSPERO), with the registration number CRD42023411141. The study followed the 2020 Preferred Reporting Items for Systematic Reviews and Meta-Analyses (PRISMA) standards.^[24]^

### 2.2. Search strategy

A systematic search of the following online databases was carried out for test reports that were eligible for inclusion: PubMed, Web of Science, Scopus, EMBASE, Cochrane, Medline, and CINAHL Database. The search period was from the creation of the database to April 2023. In addition, study references were included retrospectively to supplement access to relevant literature. When we started searching, we used the following keywords: “Internet,” “Mindfulness,” “Neoplasms,” “randomized controlled trial.” The particular search terms were created using Medical Subject Headings and thesaurus phrases. The full search query may be seen in the Supplementary Materials 1, Supplemental Digital Content, http://links.lww.com/MD/O590.

### 2.3. Inclusion criteria

We adopted the Population, Intervention, Comparison, and Study Design (PICOS) framework^[25]^ to serve as a guide for the development and establishment of eligibility criteria for this study (see Supplementary Materials 2, Supplemental Digital Content, http://links.lww.com/MD/O592). Studies had to meet the following criteria to be included in the systematic review and meta-analysis: (1) includes patients with cancer who are over the age of 18; (2) used MBIs with mindfulness as the main component, as opposed to as a subcomponent of a program (e.g., acceptance and commitment therapy), and included formal meditation homework; (3) online delivery of interventions through a web-based platform or application; (4) the use of an active control group (which receives a different type of intervention) or a non-active control group (which does not receive any intervention); (5) validated instruments assessing 1 or more mental health outcomes; (6) the study was designed as a randomized controlled trial; (7) it was available in English.

Studies were excluded if: (1) the full articles could not be located; (2) the data used to calculate the effect size for the meta-analysis were not reported in the study; and (3) the study had seemingly conflicting information.

### 2.4. Study selection

Two reviewers (G-AX and H-YX) independently performed the literature search and screened the titles/abstracts and full text of candidate articles based on the inclusion/exclusion criteria. In case of disagreement, discussions were held with the corresponding authors to reach a consensus.

### 2.5. Data extraction

The data extraction process was a standardized process by 1 reviewer (G-AX), and a second reviewer (H-YX) then checked the accuracy of the data. Differences of opinion were the subject of discussion and agreement. Relevant extracted data includes: first author, publication year, study country, cancer type, sample size per grope, gender (% women), age, type of MBI, control group type (active or inactive), method of delivery (e.g., video-conferencing, website or mobile application), guidance (with or without), number of sessions, duration of intervention, time of measurement (postintervention or follow-up), type of instruments used to assess the primary outcome (anxiety, depression and stress) and secondary outcomes (cancer-related quality of life, sleep disturbance and fatigue severity), and measurement time. If certain data could not be extracted or were not clearly stated in the study, the relevant author was contacted via e-mail to obtain the required information.

### 2.6. Appraisal of methodical quality

The quality of the included RCTs was assessed by 2 reviewers (G-AX and X-LC) independently of each other using the Cochrane Handbook for Systematic Reviews of Interventions 5.1.0.^[26]^ These included (1) random sequence generation, (2) allocation concealment, (3) blinding of participants and investigators, (4) blinding of outcome assessments, (5) incomplete outcome data, (6) selective outcome reporting, and (7) other biases. Each subject of the included studies was rated by the Cochrane Handbook as having a low risk of bias, a high risk of bias, or an unknown risk of bias. Each aspect of the included studies was categorized as either low risk of bias, high risk of bias, or unclear risk of bias in accordance with the Cochrane Handbook. Whenever a disagreement arises, resolution is achieved through discussion.

### 2.7. Quality of evidence assessment

The Grading of Recommendations, Assessment, Development and Evaluation (GRADE) were used to assess the quality of evidence of the included studies.^[27]^ With potential scores of high, moderate, low, and extremely low, this evaluation goes beyond the risk of bias and includes 5 assessment domains: bias risk, outcome inconsistency, indirectness, imprecision, and publishing bias.^[28]^ This assessment was carried out using GRADE pro, and each outcome’s data produced a “Summary of Findings Table” with corresponding footnotes explaining any judgments to decrease the quality of the evidence.^[27]^

### 2.8. Statistical analysis

The Cochrane Collaboration’s Review Manager version 5.4 software was used. Except for fatigue severity, which is evaluated by the same instrument and a WMD is applied, we utilized the standardized mean difference (SMD) as the summary statistic for the primary and secondary outcomes. This is because the studies included various psychometric instruments to assess almost all outcomes. For each outcome, the study presented quantitative data and reported the SMD along with its corresponding 95% confidence interval (CI) as the “effect size.”^[26]^ The I^2^ statistic was used to assess the statistical heterogeneity of the studies included. Mild, moderate, and high heterogeneity were represented by I^2^ values between 0% to 25%, 25% to 50%, and >75%, respectively.^[29]^ A fixed-effects model was used when I^2^ < 50% and *P* > .1. Otherwise, a random effects model was used. If trial data could not be gathered, a narrative synthesis was conducted. We used the random effects model for all analyses since there was significant variability and variation in the interventions and features of the study populations.

## 3. Results

### 3.1. Literature search

**Figure 1.**
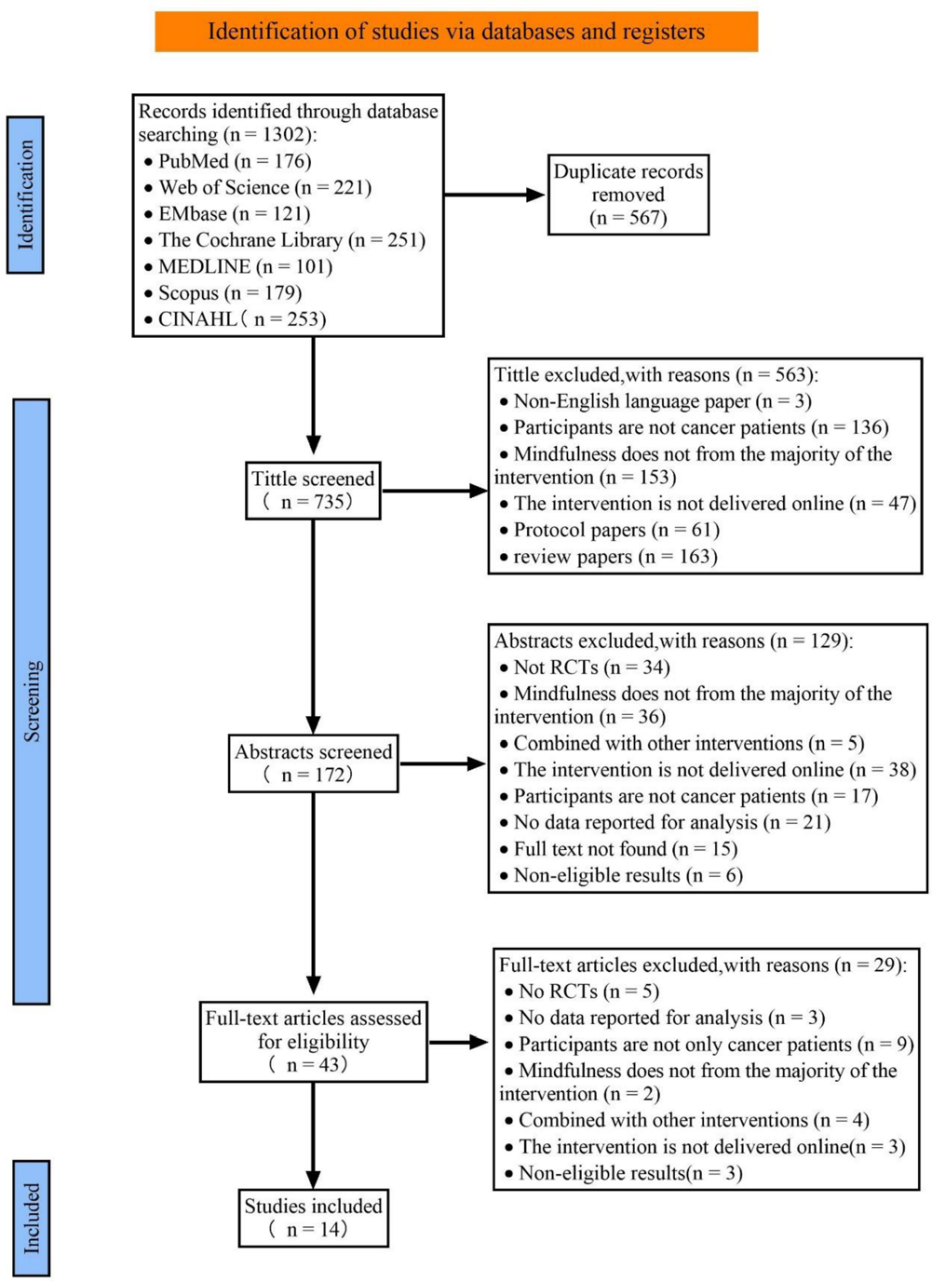
Flow chart of study selection.

The initial search found 1302 potential studies for analysis. After excluding 567 duplicates, 563 studies were eliminated based on title and 129 on abstract. These studies did not meet the inclusion criteria or have full text access. The remaining 43 articles were fully accessible. After assessing these articles based on the inclusion criteria, we ultimately included 14 RCT articles^[30–43]^ in this systematic review and meta-analysis. The detailed study selection process is shown in Figure 1.

### 3.2. Study characteristics

Table 1 summarizes the characteristics of the included trials. A total of 14 RCTs was conducted in 8 countries: Canada (n = 1), the Netherlands (n = 4), the USA (n = 1), Australia (n = 1), Denmark and Sweden (n = 1), China (n = 4) and Spain (n = 1). A total of 1316 adult individuals (average age: 45–67 years) were included in these studies, with 703 getting MBIs online and 613 serving as controls. The studies’ sample sizes ranged from 57 to 168. In all trials, women made up more than half of the study population. The studies included different types of cancer: all types (n = 5), breast (n = 5), melanoma (n = 1), breast and prostate (n = 1), liver (n = 1), and colorectal (n = 1). Three studies examined MBSR, 5 MBCT, and 6 comparisons were utilized as an Internet-based mindfulness treatment that was not MBSR or MBCT. All of the trials utilized therapies that included instruction during the intervention. Individual coaching and feedback (e.g., answering questions, feedback on tasks, positive reinforcement) were supplied by email, an encrypted website, telephone, and/or WeChat in 5 of these comparisons. In terms of delivery method, the interventions were primarily administered through a website (n = 8). Other methods included a mobile application (n = 2), videoconferencing (n = 3), and WeChat (n = 2). The MBIs varied from 1.5 to 4 hours per week, and the intervention duration ranged from 4 to 8 weeks. An inactive control condition was employed in 8 RCTs, all of which were waiting list groups. The other 6 RCTs utilized an active control condition in which participants either received psychoeducation (n = 3) or health education (n = 3). The 8 trials looked at post-intervention follow-up periods ranging from 2 to 34 weeks. The main outcome variables included stress in 4 comparisons, anxiety in 6 comparisons, and depression in 6 comparisons. Quality of life, sleep quality, and level of exhaustion were secondary end measures that were compared among 6 comparisons. Each instrument has strong psychometric characteristics.

**Table 1:** Characteristics of included studies.

Authors, year/country	BSZ (iMBIs/CG)	%F	Age, mean iMBIs/CG (SD)	Cancer types	Intervention	Guidance	Deliver mode	N hours per week, duration in weeks	Control group	Measurements	Outcome measured
Zernicke et al 2014/Canada^[43]^	62 (30/32)	72.6	58.0 (8.2）/58.0 (13.0）	All types	MBSR	With	Website	1.5 hours, 8 weeks	Inactive (waitlist)	Pre, post, 8 weeks follow up	Stress: CSOSI; depression/anxiety: POMS
Bruggeman-Everts et al 2017/Netherlands^[30]^	105 (55/50)	75.2	51.36 (12.04)/56.54 (8.43)	All types	MBCT	With	Website	4 hours, 9 weeks	Active (psycho-education)	Pre, post, 2 weeks follow up, 6-month follow up	Fatigue severity: CIS-FS; depression/anxiety: HADs
Compen et al 2018&Compen et al 2020/Netherlands^[32,33]^	168 (90/78)	85.1	52.4 (10.7)/50.4 (9.9)	All types	MBCT	With	Website	2.5 hours, 8 weeks	Inactive (waitlist)	Pre, post	Depression/anxiety: HADs; quality of life: SF-12 Physical, EQ-5D
Rosen, K D et al 2018/USA^[38]^	112 (57/55)	100	51.40 (10.73)/50.4 (9.9)	Breast	MBIs	With	Mobile application	8 weeks	Inactive (waitlist)	Pre, during, post, 12 weeks follow up	Quality of life FACT-B
Russell et al 2019/Australia^[39]^	69 (46/23)	53.6	53.5 (12.1)/53.1 (15.2)	Melanoma	MBIs	With	Website	6 weeks	Inactive (waitlist)	Pre, post	Stress: PSS
Nissen et al 2021/Denmark, Sweden^[35]^	150 (104/46)	91.3	55.11 (10.26)/56.22 (9.22)	Breast and prostate	MBCT	With	Website	10 weeks	Inactive (waitlist)	Pre, during, post, 34 weeks follow up	Anxiety: STAI-Y; depression: BDI-Ⅱ; stress: PSS; sleep quality: ISI
Shao et al 2021/China^[41]^	144 (72/72)	100	40.3 (7.0)/44.4 (8.2)	Breast	MBIs	With	WeChat	6 weeks	Active (health education)	Pre, post, 1-month follow up, 3-month follow up	Depression: PHQ-9; anxiety: GAD-7; sleep quality: AIS
Schellekens et al 2022/Netherlands^[40]^	75 (29/46)	73.3	53.24 (11.13)/56.15 (8.66)	All types	MBCT	With	Website	9 weeks	Active (psycho-education)	Pre, post	Fatigue severity: CIS-FS
Liu et al 2022/China^[34]^	122 (61/61)	23	54.36 (8.46)/57.02 (8.00)	Liver	MBIs	With	WeChat	6 weeks	Inactive (waitlist)	Pre, post, 1-month follow up, 3-month follow up	Depression/anxiety: HADs; sleep quality: PSQI; quality of life: FACT-Hep; stress: PSS
Wang et al 2022/China^[42]^	103 (51/52)	100	45.37 (7.59)/48.12 (8.05)	Breast	MBSR	With	Videoconferencing	2 hours, 4 weeks	Active (health education)	Pre, post, 1-month follow up	quality of life: FACT-B
Peng et al 2022/China^[36]^	57 (30/30)	100	/	Breast	MBIs	With	Videoconferencing	1.5 hours, 6 weeks	Inactive (waitlist)	Pre, post, 1-month follow up	Quality of life: EORTC-QOQ-C30
Rocamora et al 2022/China^[37]^	82 (39/43)	35.3	63.7/66.4	Colorectal	MBIs	With	Mobile application	/	Inactive (waitlist)	Pre, post, 1-month follow up	Depression/anxiety: HADs
Chang et al 2022/China^[31]^	72 (40/32)	100	53.38 (13.04)/47.21 (10.83)	Breast	MBSR	With	Website	2 hours, 6 weeks	Active (health education)	Pre, post	Depression/anxiety/stress: DASS-21;
Zernicke et al 2014/Canada^[43]^	62 (30/32)	72.6	58.0 (8.2）/58.0 (13.0）	All types	MBSR	With	Website	1.5 hours, 8 weeks	Inactive (waitlist)	Pre, post, 8 weeks follow up	Stress: CSOSI; depression/anxiety: POMS

AIS = Athens Insomnia Scale, BDI = Beck’s Depression Inventory, BSZ = baseline sample size, CG = control group, CIS-FS = Checklist Individual Strength-Fatigue Severity subscale, CSOSI = Calgary Symptoms of Stress Inventory, DASS-21 = Depression Anxiety Stress Scales-21, EORTC-QOQ-30 = European Organization for Research and Treatment of Cancer questionnaire, EQ-5D = EuroQol-5D-3L, %Fa = percentage of women in the total study population at baseline, FACT-B = Functional Assessment of Cancer Therapy-Breast version 4, FACT-Hep = Functional Assessment of Cancer Therapy-Hepatobiliary Carcinoma, GAD-7 = General Anxiety Disorder-7 item, HADS = Hospital Anxiety and Depression Scale, ISI = Insomnia Severity Index, MBCT = mindfulness-based cognitive therapy, MBIs = mindfulness-based interventions, MBSR = mindfulness-based stress reduction, PHQ-9 = Patient Health Questionnaire, Depression Module, POMS = Profile of Mood States, PSQI = Pittsburgh Sleep Quality Index, PSS = perceived stress scale, SF-12, Short Form-12, STAI-T = State-Trait Anxiety Inventory–Trait Subscale.

### 3.3. Methodological quality

**Figure 2.**
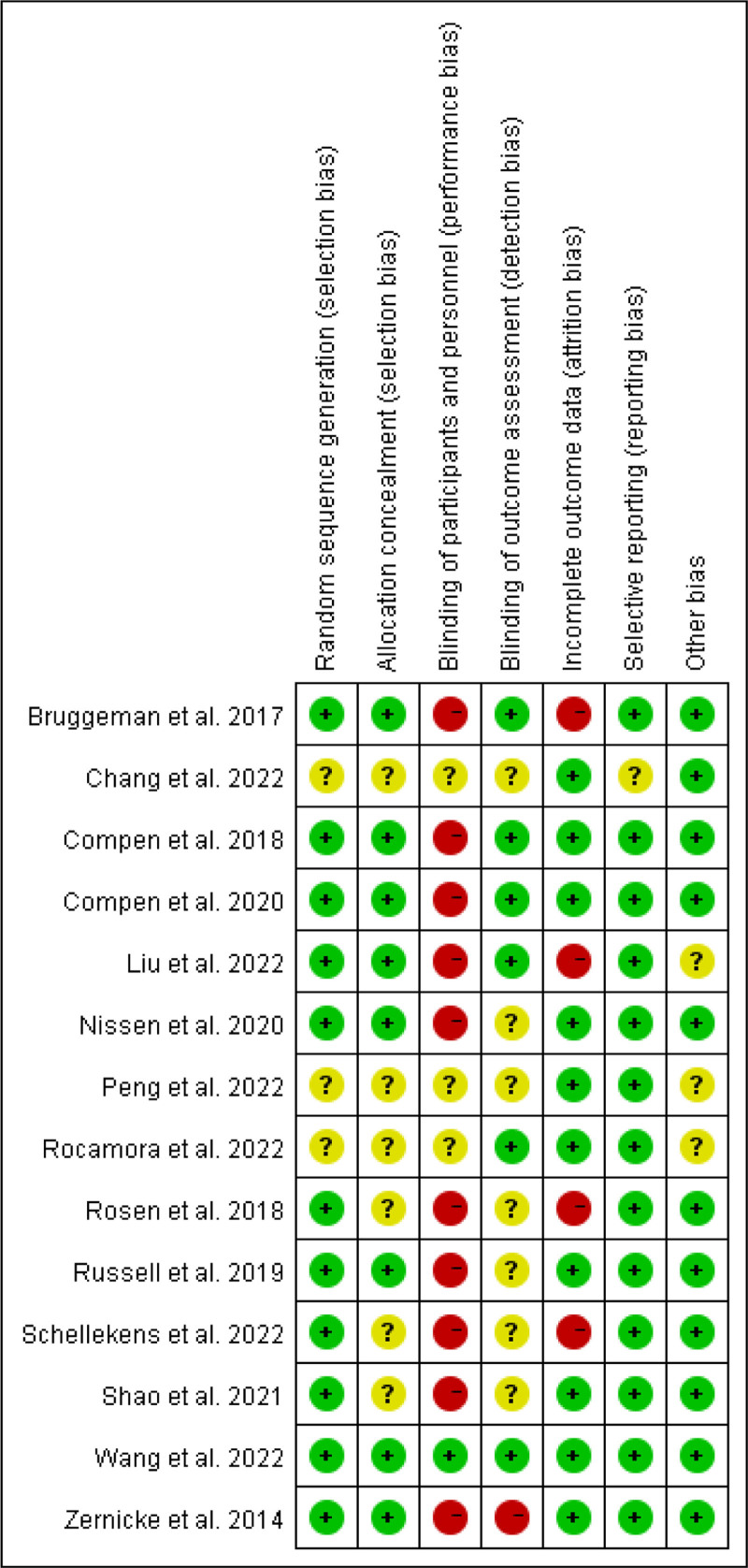
Risk of bias summary: the review authors’ assessment of each risk of bias for each included study.

The risk of bias of the included studies is summarized in Figure 2. Eleven studies used in random sequence generation, 3 studies reported incomplete information on the randomization process. Eight trials disclosed explicit allocation concealment techniques, whilst the remaining 6 trials did not. Six trials had blinded outcome assessors, while the remaining RCTs that were deemed to have ambiguous detection bias were not published. Only 1 study had blinding of participants and/or personnel, ten studies reported that blinding was not used, and other studies did not have blinding. Four trials reported partial data loss and were deemed to have a high risk of attrition bias. The remaining RCTs were assessed to have a low risk. Only 1 RCT did not report trial registration, so the trial was considered to have unclear selective reporting bias. Eleven RCTs disclosed receiving a government grant, which was evaluated as low risk, while the remaining 3 trials, for which there was no mention, were assessed as unclear for other biases (see Supplementary Materials 3, Supplemental Digital Content, http://links.lww.com/MD/O593).

### 3.4. Meta-analysis outcome

#### 3.4.1. Primary outcomes

##### 3.4.1.1. Effects on anxiety

**Figure 3.**
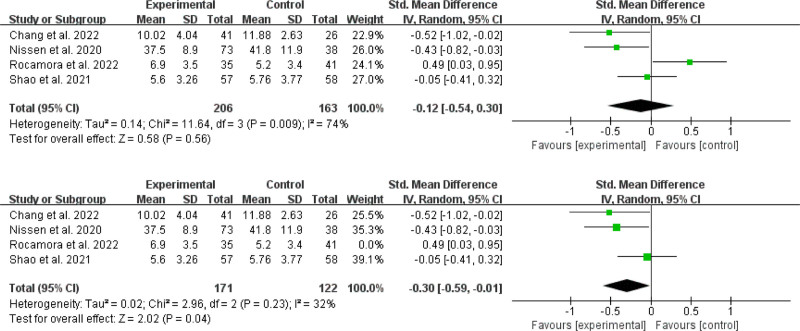
Effects on anxiety.

Four RCTs^[31,35,37,41]^ involving 206 participants who received online MBIs and 163 controls were analyzed to calculate the aggregated impact on anxiety (Fig. 3). According to the meta-analysis, online MBIs had no significant effect on anxiety when compared to the control condition [SMD = −0.12; 95% CI, −0.54 to 0.30; *P* = .56]. The result showed I^2^ = 74% and *P* = .009, showing significant statistical heterogeneity. Outliers were discovered^[37]^ by sensitivity analyses excluding 1 study at a time. We discovered a distinct effect after omitting these papers from the analysis, with SMD = -0.30 [95% CI (−0.59, −0.01), *P* = .04] and a significant reduction in heterogeneity (I^2^ = 32%, *P* = .23).

##### 3.4.1.2. Effects on depression

**Figure 4.**
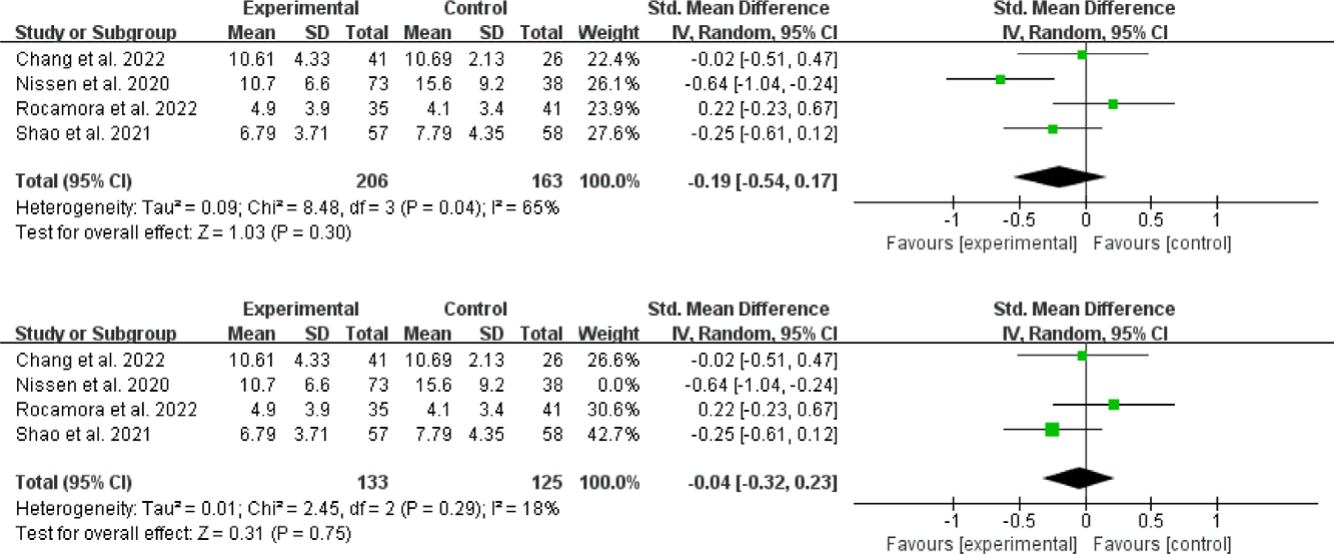
Effects on depression.

Four trials yielded a total of 206 online MBIs and 163 controls^[31,35,37,41]^ were included in the meta-analysis for depression outcomes (Fig. 4). The overall effect of online MBIs on depression was not statistically significant (SMD = −0.19; 95% CI, −0.54 to 0.17; *P* = .30). A high degree of heterogeneity was observed (*P* = .04; *I^2^* = 65%). Outliers were detected^[35]^ by sensitivity analyses excluding 1 study at a time. After removing the outlier, the pooled effect was reduced to SMD = -0.04, 95% CI (−0.32 to 0.23), although this effect remained nonsignificant (*P* = .75). Furthermore, heterogeneity decreased to a moderate level (I^2^ = 18%, *P* = .29). Three studies^[33,34,43]^ found that an online MBI intervention reduced psychological distress in cancer patients significantly. However, 1 study^[30]^ did not report whether online MBIs reduced psychological distress in cancer patients. The above 4 papers did not have sufficient data for meta-analysis.

##### 3.4.1.3. Effects on stress

**Figure 5.**
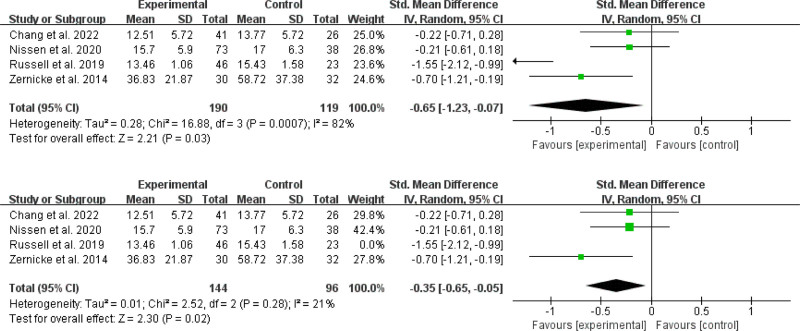
Effects on stress.

For the stress outcome, we successfully included 4 studies^[35,39,43,44]^ with 190 online MBIs and 119 controls (Fig. 5). According to the findings of the study, online MBIs were more successful than the control group in reducing stress levels (SMD = −0.65; 95% CI, −1.23 to − 0.07; *P* = .03). There was a substantial degree of heterogeneity seen (*P* = .0007; *I^2^* = 82%). One potential outlier^[39]^ was identified sensitivity analyses excluding 1 study at a time. After the outlier was removed, the pooled impact was reduced to SMD = −0.35, 95% CI (−0.65, −0.05), and there was still a statistically significant effect (*P* = .02). Furthermore, the degree of heterogeneity was lowered (*I^2^* = 21%, *P* = .28). A trial^[34]^ found that perceived stress in adults with liver cancer was reduced after a 6-week online MBI intervention.

#### 3.4.2. Secondary outcomes

##### 3.4.2.1. Effects on quality of life

**Figure 6.**
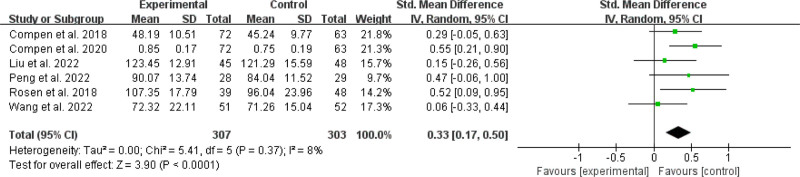
Effects on quality of life.

Six^[32–34,36,38,42]^ trials were examined for impacts on cancer-related quality of life, comprising 307 people in the online MBI intervention groups and 303 controls (Fig. 6). The results showed I^2^ = 8% and *P* = .37, with mild statistical heterogeneity. The meta-analysis found that, when compared to the control condition, the online MBIs group had a significant influence on quality of life (SMD = 0.33; 95% CI, 0.17 to 0.50; *P* < .0001).

##### 3.4.2.2. Effects on sleep quality

**Figure 7.**
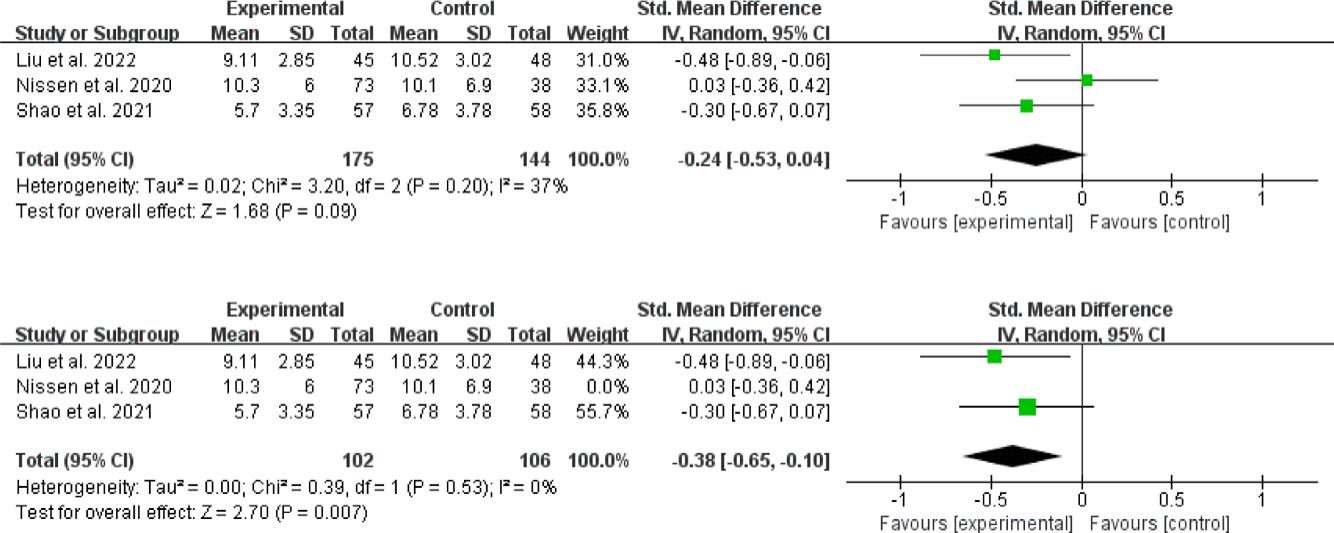
Effects on sleep quality.

Three included studies^[34,35,41]^ investigated sleep quality outcomes in 175 online MBIs and 144 control patients (Fig. 7). The meta-analysis revealed that online MBIs yielded no substantial impact on sleep quality (SMD = −0.24; 95% CI, −0.53 to 0.04; *P* = .09) in contrast to the control condition. The results were *I^2^* = 37% and *P* = .20, indicating moderate statistical heterogeneity. Sensitivity analysis using the leave-one-out method showed that 1 study^[35]^ had a significant impact on the importance of overall effect size. After exclusion of this study, the combined effect decreased to SMD = −0.38, 95% CI (−0.65, −0.10), and there was a statistically significant impact on the quality of sleep (*P* = .007). Furthermore, there was no statistical heterogeneity with a degree of heterogeneity (*I^2^* = 0%, *P* = .53).

##### 3.4.2.3. Effects on fatigue severity

**Figure 8.**
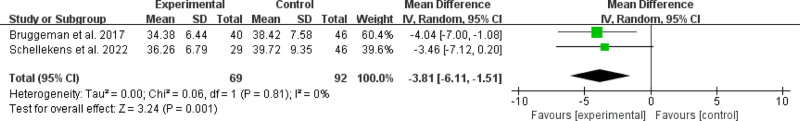
Effects on fatigue severity.

Of the fourteen RCTs, only 2^[30,40]^ reported data on fatigue severity (Fig. 8), and. The results showed that the heterogeneity was low, with *I*^2^ = 0% and *P* = .81. The meta-analysis found that online MBIs had a substantial influence on tiredness reduction when compared to the control condition (WMD = −3.81; 95% CI = −6.11, -1.51; *P* = .001).

### 3.5. Grading of recommendations, assessment, development and evaluation

**Figure 9.**
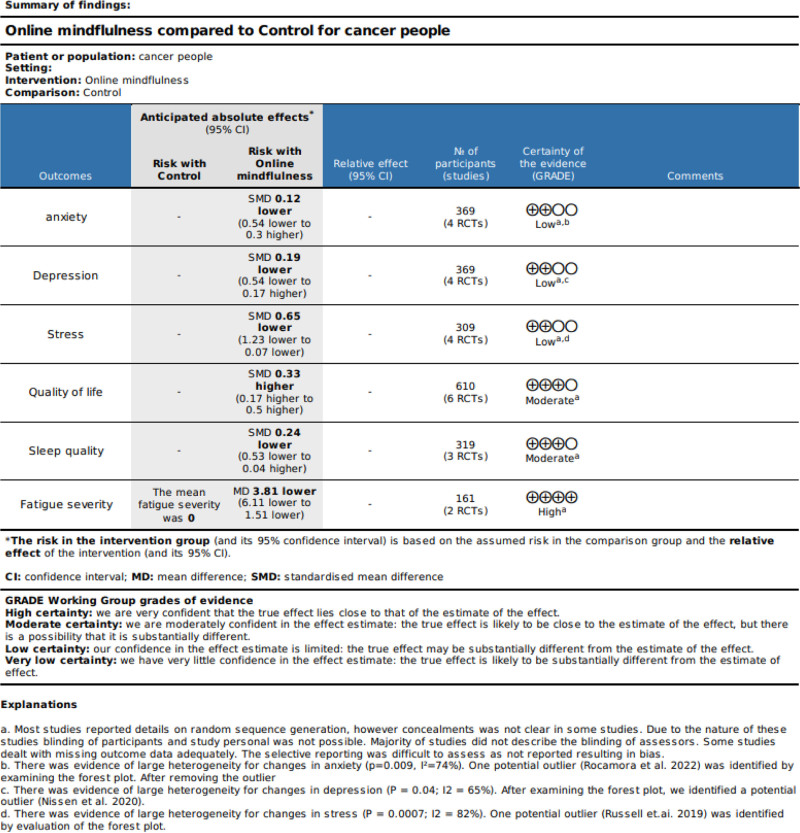
Grade

Using GRADE, the overall quality of the evidence was rated as moderate or low. This is because the overall quality of evidence for the primary outcome is rated as low, indicating a low level of confidence in the effect estimate. The level of evidence for RCTs was reduced from high to low for anxiety, depression and stress. The level of evidence for RCTs was lowered from high to moderate for quality of life, sleep quality and fatigue severity. This reclassification from high to moderate or low occurred because of significant concerns regarding the degree of variability and potential for bias. Overall, there were no serious concerns about inconsistency or indirectness (Fig. 9).

## 4. Discussion

### 4.1. Main finding

This meta-analysis included fourteen RCTs with 1316 participants that investigated the effectiveness of online MBIs on the psychological and physical health of cancer patients. The combined analyses revealed that online MBIs reduced stress and improved cancer-related quality of life while also lowering sleep disruption and exhaustion. However, we did not discover a clear, significant influence on depression. In addition, the results show that there is controversy about the efficacy of online MBIs in improving anxiety in cancer patients, and when this literature^[37]^ by Rocamora was excluded, the statistical results shifted from nonsignificance to significance. This could be due to the fact that the mean age in this study was higher (65 years) and the preoperative anxiety was lower. These factors may have limited the potential efficacy of online MBIs in less favorable conditions, thus influencing the results of the meta-analysis.

The publication dates of 9 out of 14 studies being after 2020 demonstrate the increasing attention towards this intervention type amidst the COVID-19 pandemic. Although there has been an upsurge in studies on this topic, the impact of online MBIs on the mental health of cancer patients has yet to be demonstrated. The pooled investigation revealed that online MBIs reduce anxiety and stress, demonstrating that they have a positive impact on mental health issues. Our study’s findings align with those of a previous publication on MBIs in individuals with physical health condition.^[21]^ The results of this study may be helpful in reducing depression, anxiety, and stress.

However, the types of MBIs used and the length of time they were administered vary, and earlier meta-analyses did not only concentrate on cancer patients. As a result, this meta-analysis is the first to show how online MBIs affect cancer patients’ mental health. Cancer patients not only have to endure the multiple pains caused by the disease itself and its treatment, but also have to deal with many problems such as role changes, financial burdens, and fear of recurrence, leading to significant psychosocial problems and a range of psychological disorders,^[45]^ and data^[46]^ show that about one-third of cancer patients suffer from psychological disorders. For cancer patients, sensations of stress and anxiety are a significant source of anguish and psychological suffering.^[47]^ There is increasing concern regarding the mental health of cancer patients. If the disease’s transient psychological hurdles are not resolved, it may lead to long-term mental and physical health problems, including suicidal ideation. The study results may suggest a convenient tool that healthcare professionals could utilize to handle mental health issues in cancer patients.

We found that the impact of online MBIs on anxiety and stress among cancer participants was not as significant as its impact on adult participants in this meta-analysis^[22]^ (including non-cancer participants). This might be linked to increased familiarity with online and mobile applications from a young age, which provides an advantage in practicing online MBIs. The average age of participants included in this review was over 40 years old, which is higher than in another review^[22]^ (average age: 22–36 years). However, it was noted in this meta-analysis that the effect sizes of online MBIs for anxiety and stress were generally larger among participants with cancer when compared to those with other physical health conditions^[21]^ (including non-cancer participants) in previous research. This may be related to the fact that people have become more adept at using electronic devices in recent years. Nine of the fourteen studies in this review were published after 2020, and all the studies in another review^[21]^ were published before 2019.

Our investigation found no substantial effect on depression. Although 3 RCTs found that online MBIs were effective in lowering depression, this meta-analysis found no statistically significant reduction in depression. Online MBIs were found to have a significant effect on depression in some review.^[15,21,22]^ However, one study^[48]^ found that the effect of online MBIs on depression was not significant in people with a diagnosis of depression. The divergent outcomes of the meta-analyses can be clarified by various factors, such as the delivery method of the program or the sampling of distinct groups and physical health afflictions.^[49]^ In addition, 3 studies included in this evaluation revealed that an online MBI intervention had a substantial effect on lowering depression, but they were unable to be included in the analysis due to a lack of data on depression ratings. The quantity of studies analyzing depression is limited and the heterogeneity is considerable. Therefore, it is suggested that high-quality, multicenter studies with large samples be conducted to assess the impact of online MBIs on depression.

In addition, online MBIs were found to improved cancer-related quality of life, and reduced sleep disturbance and fatigue. In terms of quality of life, previous systematic reviews have produced mixed results regarding the impact of mindfulness on this variable. While 1 study^[6]^ found no significant effect, another^[11]^ reported a small yet significant effect. Whether this variability in outcome measures utilized in the included studies is attributable to other variables remains uncertain, and additional research on this symptom may be necessary. Sleep quality had a noteworthy impact after pooling data for this review. This is also reflected in previous reviews. According to Zhang,^[50]^ women with breast cancer reported some improvements in sleep after a mindfulness intervention. However, these results were not statistically significant, which contrasts with the findings of another study.^[6]^ Few prior systematic reviews have evaluated sleep as an objective of the analysis. In this review, only 3 post-intervention studies investigated this factor as an outcome. Accordingly, it is challenging to discern the effect of mindfulness on sleep. This large effect of mindfulness on cancer-related fatigue (WMD = -3.81) is consistent with other reviews^[51–53]^ showing similar effects for fatigue. One research study^[54]^ discovered that mindfulness had the most significant impact on fatigue related to cancer both directly and indirectly by reducing or avoiding anxiety, depression, and sleep disruptions.

In the meta-analysis at hand, the impact of MBI on anxiety and depression appeared to be less impactful in comparison to previously conducted studies on face-to-face MBI.^[6,11,55]^ Donkin^[56]^ suggest that the lower adherence rate associated with online MBIs may explain why they are less effective compared to face-to-face MBIs. Adherence is a prevalent issue in online psychological interventions that can diminish their effectiveness.^[21]^ Toivonen^[57]^ found that lower adherence to web-based MBIs may be linked to the relatively lower social support associated with such interventions. This could partly explain the less-than-optimal mental health outcomes, including anxiety and depression.

As previous reviews^[51]^ has shown online interventions for mental health problems have proliferated. COVID-19 has been a catalyst for the use of online health management services and has been largely well received,^[58]^ although it still has its issues such as poor bandwidth and users’ technical skills.^[59]^ Nevertheless, investigating the use of these platforms for delivering mindfulness interventions would be advantageous as it would enable scalability and facilitate reaching a larger population, especially those residing in hard-to-reach rural areas.^[60]^ Online therapies are expected to offer more benefits than traditional in-person therapy in terms of accessibility, acceptance, scalability, and cost-effectiveness.^[61]^ Online therapies designed to address the psychological issues of cancer patients have the potential to alleviate concerns about stigma, time constraints, and unfamiliarity with the healthcare system.^[62]^ Furthermore, widespread smartphone use and competency with blended learning methods suggest that the majority of adults are suitable with an online, digital health support system.^[63]^ Other global factors, such as the COVID-19 pandemic, require social distancing and prolonged periods indoors to limit the virus’s transmission. As a result, an efficient online psychological intervention for cancer patients appears particularly fitting and urgent.

Additionally, the cost of treatment could impact patients’ choices to pursue mental health care.^[62]^ One study^[64]^ reported that many cancer patients complained of inconvenience due to the time spent seeking treatment. Moreover, transportation and treatment scheduling also influence patients’ seeking of treatment.^[65]^ Ultimately, online-based therapy proves feasible and highly acceptable, offering a more convenient method of intervention that could help overcome economic and adherence barriers.^[66,67]^

This review includes an examination of frequently utilized MBI programs, such as MBSR and MBCT,^[68]^ which are psychological level interventions. Per a broad definition, select traditional Chinese mind-body integration training (IBMT) programs such as taijiquan, Baduanjin, and qigong, have been grouped within MBI as well.^[69]^ Studies have demonstrated the potential for exercise therapies to confer physical and mental health benefits to practitioners.^[68]^ In light of these findings, further research ought to examine the modifications necessary to render these programs available online to cancer patients.

### 4.2. Strengths and limitations

To our knowledge, this marks the initial comprehensive systematic review and meta-analysis of online MBIs for individuals diagnosed with cancer who have mental health apprehensions. We thoroughly examined the evidence from the included RCTs. This method is widely considered the optimal and recommended approach for evaluating the effects of interventions.^[70]^ Furthermore, the meta-analyses typically included a large and geographically diverse set of samples from 8 different countries (China, the United States, Canada, the Netherlands, Australia, Denmark, Sweden, and Spain) and 4 continents (Europe, the Americas, Asia, and Oceania), which may improve the generalizability of the research findings. We were able to establish the precise physical consequences of online mindfulness-based therapies for cancer patients in addition to the psychological concerns. We strictly adhered to the PRISMA guidelines (PRISMA 2020 checklist). We carried out comprehensive literature searches across numerous databases, including investigation of gray literature to prevent overlooking unpublished studies. Moreover, we evaluated the potential for bias and condensed the accessible evidence using the GRADE method.

Although we made every effort to ensure that the quality of the trials was as high as possible, we also recognized that there were some limitations.

Firstly, the majority of the included studies contain methodological flaws or other flaws that restrict the strength and feasibility of the clinical data. The meta-analysis described in this paper revealed significant variability, which might be attributed to varied degrees of methodological quality, participant characteristics, interventions, and outcome measures. As a result, more rigorous studies adopting higher standards of RCT technique are required to determine the impact of online MBIs on the mental health of cancer patients. Secondly, there was significant variability in the features of the studies, including the sample, the measures utilized for assessing outcomes, and notably, the protocol and approach for administering the MBIs. Although the random effects model and SMD were used to minimize the possible effects of variability, our results should be interpreted with caution. These limitations are well reflected in our GRADE rating of the evidence that is available to us. Thirdly, our bibliographic search was limited to publications in English. In addition, unpublished data was not searched for. The inability to identify other relevant trials may have been limited by these 2 factors. What’s more, the limitation of this meta-analysis was the small number of eligible RCTs, which were only fourteen in total. Because this may affect the statistics’ reliability and validity, it may explain why changes in some analyses did not approach statistical significance. Fourth, analyses for certain covariates were not feasible as a result of the limited number of studies included in each subgroup. Future studies and researchers could consider these questions and design larger studies to demonstrate and improve the usefulness of online MBIs for improving different mental health problems.

### 4.3. Implications for practice

Based on the evidence available, this meta-review concludes that online MBIs are beneficial in reducing psychological distress (anxiety and stress) and a range of physical symptoms (quality of life, sleep disturbance and fatigue) in cancer patients and survivors. Although the effects are generally small, the lower cost and easy accessibility of online MBIs may make them a viable alternative to promote mental and physical health when a cancer patient returns home from hospital treatment. Given the increasing interest and possible usefulness of online MBIs for cancer patients in clinical settings, further study is needed to determine the greatest value of such interventions and the factors that influence their effectiveness.

## Author contributions

**Conceptualization:** Lichun Xu, Aixuan Guan.

**Data curation:** Aixuan Guan, Yuxin Huang.

**Formal analysis:** Lichun Xu, Aixuan Guan.

**Project administration:** Lichun Xu.

**Software:** Aixuan Guan, Yuxin Huang.

**Supervision:** Lichun Xu.

**Validation:** Yuxin Huang.

**Writing – original draft:** Aixuan Guan.

**Writing – review & editing:** Lichun Xu, Aixuan Guan, Yuxin Huang.

## Supplementary Material

SUPPLEMENTARY MATERIAL
