# Supplementary material for: Effects of online mindfulness-based interventions on mental and physical health outcomes in cancer patients: A systematic review and meta-analysis of randomized controlled trials
Source: Medicine (Baltimore). 2025 Mar 21;104(12):e41870. doi: 10.1097/MD.0000000000041870 (PMC11936609; doi:10.1097/MD.0000000000041870)
Supplement: SUPPLEMENTARY MATERIAL [file medi-104-e41870-s001.docx]

**SUPPLEMENTARY MATERIALS**

Effects of online mindfulness-based interventions on mental and physical health outcomes in cancer patients: A systematic review and meta‐analysis of randomized controlled trials

Lichun Xu^a*^, Aixuan Guan^b*^, Yuxin Huang^a^

（ a Department of Nursing, Zhongshan Hospital Affiliated to Xiamen University, Xiamen 361004, China; b Department of Respiratory and Critical Care Medicine, Longyan First Hospital Affiliated to Fujian Medical University, Longyan, 364000, China)

*These authors contributed equally to this work.

**S1 Table. Search strategy**

| **Database** | **Keyword** |
| --- | --- |
| **PubMed** | #1 “Online Systems”[MeSH Terms] OR “online”[All Fields] OR “on-line”[All Fields] OR “ehealth”[All Fields] OR “e-health”[All Fields] OR “virtual*”[All Fields] OR “web”[All Fields] OR “webs”[All Fields] OR “website*”[All Fields] OR “Internet”[MeSH Terms] OR “internet*”[All Fields] OR “app”[All Fields] OR “apps”[All Fields] OR “application*”[All Fields] OR “mobile*”[All Fields] OR “mhealth”[All Fields] OR “m-health”[All Fields] OR “Wechat”[All Fields]  #2 “Mindfulness”[MeSH Terms] OR “mindful*” [All Fields]  #3 “Neoplasms” [MeSH Terms] OR “Tumor” [All Fields] OR “Neoplasm” [All Fields] OR “Tumors” [All Fields] OR “Neoplasia” [All Fields] OR “Neoplasias” [All Fields] OR “Cancer”[All Fields] OR “Cancers” [All Fields] OR “Malignant Neoplasm” [All Fields] OR “Malignancy” [All Fields] OR “Malignancies”[All Fields] OR “Malignant Neoplasms” [All Fields] OR “Neoplasm， Malignant” [All Fields] OR “Neoplasms, Malignant” [All Fields] OR “Benign Neoplasms” [All Fields] OR “Benign Neoplasm” [All Fields] OR “Neoplasms, Benign” [All Fields] OR “Neoplasm, Benign” [All Fields]  #4 “controlled clinical trial”[MeSH Terms] OR “randomized controlled trial”[MeSH Terms] OR “random allocation”[MeSH Terms] OR “RCT”[All Fields] OR “random*”[All Fields] OR “control*”[All Fields] OR “trial*”[All Fields]  #5 #1 AND #2 AND #3 AND #4 |
| **Web of Science** | #1 All Fields = (“Internet” OR “computer” OR “website” OR “mobile health” OR “electronic health” OR “wechat” OR “application” OR “online” OR “web-based” OR “mHealth” OR “web”)  #2 All Fields = (“Mindfulness” OR “mindful*”)  #3 All Fields = (“Neoplasms” OR “Tumor” OR “Neoplasm” OR “Tumors” OR “Neoplasia” OR “Neoplasias” OR “Cancer” OR “Cancers” OR “Malignant Neoplasm” OR “Malignancy” OR “Malignancies” OR “Malignant Neoplasms” OR “Neoplasm, Malignant” OR “Neoplasms, Malignant” OR “Benign Neoplasms” OR “Benign Neoplasm” OR “Neoplasms, Benign” OR “Neoplasm, Benign”)  #4 All Fields = (“randomized controlled trial” OR “randomized” OR “placebo” OR “RCT” OR “random*” OR “control*” OR “trial*”)  #5 #1 AND #2 AND #3 AND #4 |
| **Scopus** | #1 TITLE-ABS-KEY (“Internet” OR “computer” OR “website” OR “mobile health” OR “electronic health” OR “wechat” OR “application” OR “online” OR “web-based” OR “mHealth” OR “web”)  #2 TITLE-ABS-KEY (“Mindfulness” OR “mindful*”)  #3 TITLE-ABS-KEY (“Neoplasms” OR “Tumor” OR “Neoplasm” OR “Tumors” OR “Neoplasia” OR “Neoplasias” OR “Cancer” OR “Cancers” OR “Malignant Neoplasm” OR “Malignancy” OR “Malignancies” OR “Malignant Neoplasms” OR “Neoplasm, Malignant” OR “Neoplasms, Malignant” OR “Benign Neoplasms” OR “Benign Neoplasm” OR “Neoplasms, Benign” OR “Neoplasm, Benign”)  #4 TITLE-ABS-KEY ( “randomized controlled trial” OR “randomized” OR “placebo” OR “RCT” OR “random*” OR “control*” OR “trial*”)  #5 #1 AND #2 AND #3 AND #4 |
| **Embase** | #1 All Fields = (“Internet” OR “computer” OR “website” OR “mobile health” OR “electronic health” OR “wechat” OR “application” OR “online” OR “web-based” OR “mHealth” OR “web”)  #2 All Fields = (“Mindfulness” OR “mindful*”)  #3 All Fields = (“Neoplasms” OR “Tumor” OR “Neoplasm” OR “Tumors” OR “Neoplasia” OR “Neoplasias” OR “Cancer” OR “Cancers” OR “Malignant Neoplasm” OR “Malignancy” OR “Malignancies” OR “Malignant Neoplasms” OR “Neoplasm, Malignant” OR “Neoplasms, Malignant” OR “Benign Neoplasms” OR “Benign Neoplasm” OR “Neoplasms, Benign” OR “Neoplasm, Benign”)  #4 All Fields = (“randomized controlled trial” OR “randomized” OR “placebo” OR “RCT” OR “random*” OR “control*” OR “trial*”)  #5 #1 AND #2 AND #3 AND #4 |
| **Cochrane** | #1 Title Abstract Keyword (“Internet” OR “computer” OR “website” OR “mobile health” OR “electronic health” OR “wechat” OR “application” OR “online” OR “web-based” OR “mHealth” OR “web”)  #2 Title Abstract Keyword (“Mindfulness” OR “Mindfulness-based cancer recovery” OR “Mindfulness decompression training”)  #3 Title Abstract Keyword (“Neoplasms” OR “Tumor” OR “Neoplasm” OR “Tumors” OR “Neoplasia” OR “Neoplasias” OR “Cancer” OR “Cancers” OR “Malignant Neoplasm” OR “Malignancy” OR “Malignancies” OR “Malignant Neoplasms” OR “Neoplasm, Malignant” OR “Neoplasms, Malignant” OR “Benign Neoplasms” OR “Benign Neoplasm” OR “Neoplasms, Benign” OR “Neoplasm, Benign”)  #4 Title Abstract Keyword ( “randomized controlled trial” OR “randomized” OR “placebo” OR “RCT” OR “random*” OR “control*” OR “trial*”)  #5 #1 AND #2 AND #3 AND #4 |
| **Medline** | #1 All Fields = (“Internet” OR “computer” OR “website” OR “mobile health” OR “electronic health” OR “wechat” OR “application” OR “online” OR “web-based” OR “mHealth” OR “web”)  #2 All Fields = (“Mindfulness” OR “mindful*”)  #3 All Fields = (“Neoplasms” OR “Tumor” OR “Neoplasm” OR “Tumors” OR “Neoplasia” OR “Neoplasias” OR “Cancer” OR “Cancers” OR “Malignant Neoplasm” OR “Malignancy” OR “Malignancies” OR “Malignant Neoplasms” OR “Neoplasm, Malignant” OR “Neoplasms, Malignant” OR “Benign Neoplasms” OR “Benign Neoplasm” OR “Neoplasms, Benign” OR “Neoplasm, Benign”)  #4 All Fields = (“randomized controlled trial” OR “randomized” OR “placebo” OR “RCT” OR “random*” OR “control*” OR “trial*”)  #5 #1 AND #2 AND #3 AND #4 |
| **CINAHL** | #1 (MH “Online Systems+”) OR (MH “Internet+”) OR (MM “Internet-Based Intervention”) OR “online” OR “computer” OR “website” OR “mobile health” OR “electronic health” OR “wechat” OR “application” OR “online” OR “web-based” OR “mHealth” OR “web”  #2 (MM “Mindfulness”) OR “mindful*”  #3 (MM “Neoplasms”) OR “Tumor” OR “Neoplasm” OR “Tumors” OR “Neoplasia” OR “Neoplasias” OR “Cancer” OR “Cancers” OR “Malignant Neoplasm” OR “Malignancy” OR “Malignancies” OR “Malignant Neoplasms” OR “Neoplasm, Malignant” OR “Neoplasms, Malignant” OR “Benign Neoplasms” OR “Benign Neoplasm” OR “Neoplasms, Benign” OR “Neoplasm, Benign”  #4 (MH “Clinical Trials+”) OR (MH “Randomized Controlled Trials+”) OR “randomized” OR “placebo” OR “RCT” OR “random*” OR “control*” OR “trial*”  #5 #1 AND #2 AND #3 AND #4 |
