# Supplementary material for: Effects of online mindfulness-based interventions on mental and physical health outcomes in cancer patients: A systematic review and meta-analysis of randomized controlled trials
Source: Medicine (Baltimore). 2025 Mar 21;104(12):e41870. doi: 10.1097/MD.0000000000041870 (PMC11936609; doi:10.1097/MD.0000000000041870)
Supplement: SUPPLEMENTARY MATERIAL [file medi-104-e41870-s002.docx]

**SUPPLEMENTARY MATERIALS**

Effects of online mindfulness-based interventions on mental and physical health outcomes in cancer patients: A systematic review and meta‐analysis of randomized controlled trials

Lichun Xu^a*^, Aixuan Guan^b*^, Yuxin Huang^a^

（ a Department of Nursing, Zhongshan Hospital Affiliated to Xiamen University, Xiamen 361004, China; b Department of Respiratory and Critical Care Medicine, Longyan First Hospital Affiliated to Fujian Medical University, Longyan, 364000, China)

*These authors contributed equally to this work.

**S2 Table. PICOS framework**

| **Components of PICOS** | **Definition** |
| --- | --- |
| **P**opulation | Adults aged 18 years and older |
| **I**ntervention | Online MBIs (including MBSR or MBCT) |
| **C**omparison | Active or inactive control group |
| **O**utcome | (1) Primary outcome: to reduce symptoms of depression, anxiety, stress; (2) Secondary outcome: improvement in quality of life, sleep quality, fatigue severity. |
| **S**tudy Design | RCT conducted during the period of the cancer patients |

**MBI**, mindfulness-based intervention; **MBCT**, mindfulness-based cognitive therapy; **MBSR**; mindfulness-based stress reduction; **PICOS**, Population, Intervention, Comparison, Outcome, and Study Design; **RCT**, randomized controlled trial
