# Supplementary material for: Effects of online mindfulness-based interventions on mental and physical health outcomes in cancer patients: A systematic review and meta-analysis of randomized controlled trials
Source: Medicine (Baltimore). 2025 Mar 21;104(12):e41870. doi: 10.1097/MD.0000000000041870 (PMC11936609; doi:10.1097/MD.0000000000041870)
Supplement: SUPPLEMENTARY MATERIAL [file medi-104-e41870-s003.docx]

**SUPPLEMENTARY MATERIALS**

Effects of online mindfulness-based interventions on mental and physical health outcomes in cancer patients: A systematic review and meta‐analysis of randomized controlled trials

Lichun Xu^a*^, Aixuan Guan^b*^, Yuxin Huang^a^

（ a Department of Nursing, Zhongshan Hospital Affiliated to Xiamen University, Xiamen 361004, China; b Department of Respiratory and Critical Care Medicine, Longyan First Hospital Affiliated to Fujian Medical University, Longyan, 364000, China)

*These authors contributed equally to this work.

**S3 Fig. Result of quality assessment of included studies using the Risk of Bias 2 tool**

**
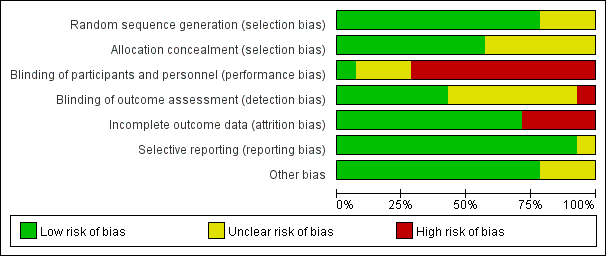
**

**
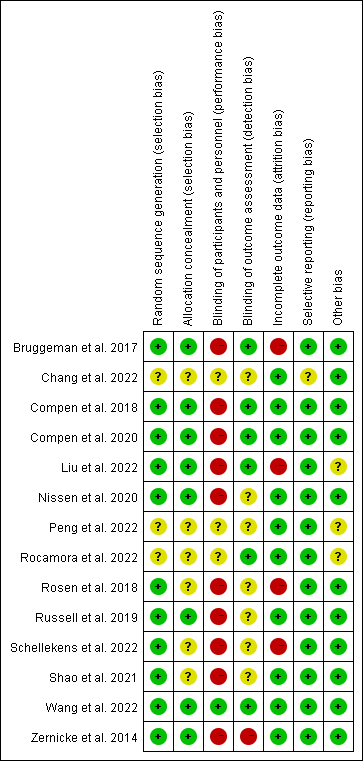
**
